# Acute Appendicitis Complicated by Pylephlebitis: A Case Report

**DOI:** 10.1155/2013/627521

**Published:** 2013-11-07

**Authors:** Ricardo Castro, Teresa Fernandes, Maria I. Oliveira, Miguel Castro

**Affiliations:** Department of Radiology, Hospital de S. Joao, Alameda Prof. Hernâni Monteiro, 4200-319 Porto, Portugal

## Abstract

Pylephlebitis is defined as septic thrombophlebitis of the portal vein. It is a rare but serious complication of an intraabdominal infection, more commonly diverticulitis and appendicitis. It has an unspecific clinical presentation and the diagnosis is difficult. The authors report a case of a 21-year-old man with acute appendicitis complicated by pylephlebitis. The diagnosis was made with contrast enhanced CT.

## 1. Introduction

Pylephlebitis refers to infective suppurative thrombosis of the portal vein. It represents a rare but serious complication of an intraabdominal inflammatory process [1]. The diagnosis is difficult due to its nonspecific clinical presentation. Mortality and morbidity remain elevated, because it may be complicated by hepatic abscesses or mesenteric veins occlusion, leading to bowel ischemia and infarction [2]. However, if a prompt diagnosis is achieved, it can be treated with early and aggressive interventions. 

The case presented here documents the CT findings of a case of acute appendicitis complicated by superior mesenteric and portal vein thrombophlebitis. 

## 2. Case Report

A 20-year-old Caucasian male, previously healthy, presented to our hospital with a 10-day history of abdominal pain, more intense in the right lower quadrant. He complained from fever and worsening of the pain in the last 3 days. He denied bloody stools, nausea, or vomiting. The only relevant finding on the physical examination was tenderness in the right lower quadrant. Initial laboratory tests showed increased white blood cell counts (18.97 × 10^9^/L) and increased C-reactive protein (306.2 mg/L). Liver enzyme levels were elevated (aspartate aminotransferase—58 IU/L; alanine aminotransferase—59 IU/L; gamma-glutamyltransferase—169 IU/L; alkaline phosphatase—127 IU/L). 

**Figure 1:**
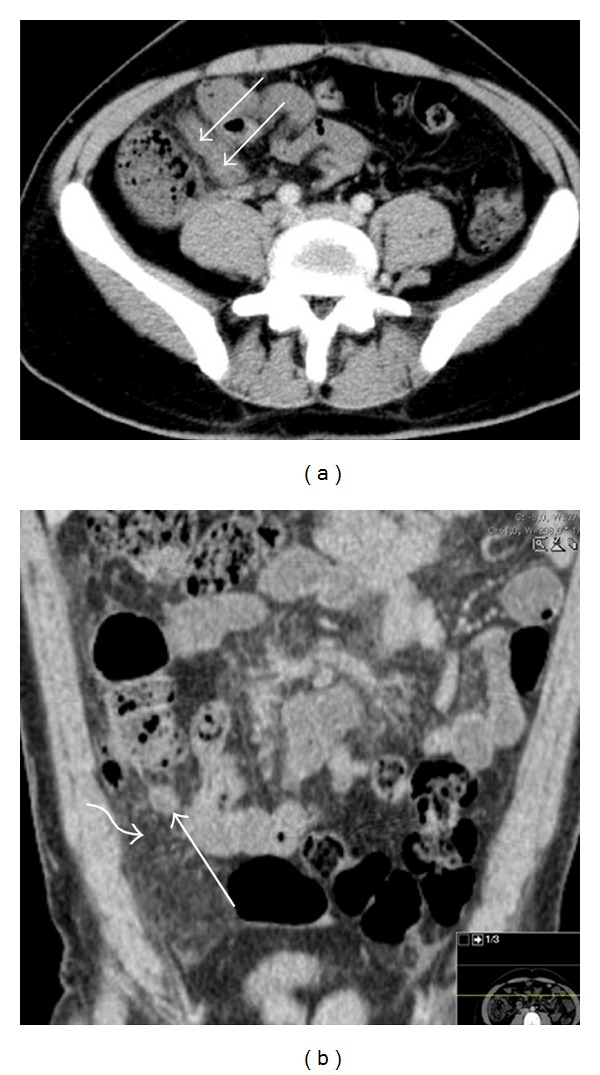
Axial contrast-enhanced (a) and coronal reconstruction (b) CT images obtained at the level of the lower abdomen show an enlarged and thick-walled appendix (arrows), with evidence of stranding of the surrounding mesenteric fat (curved arrow in (b)).

**Figure 2:**
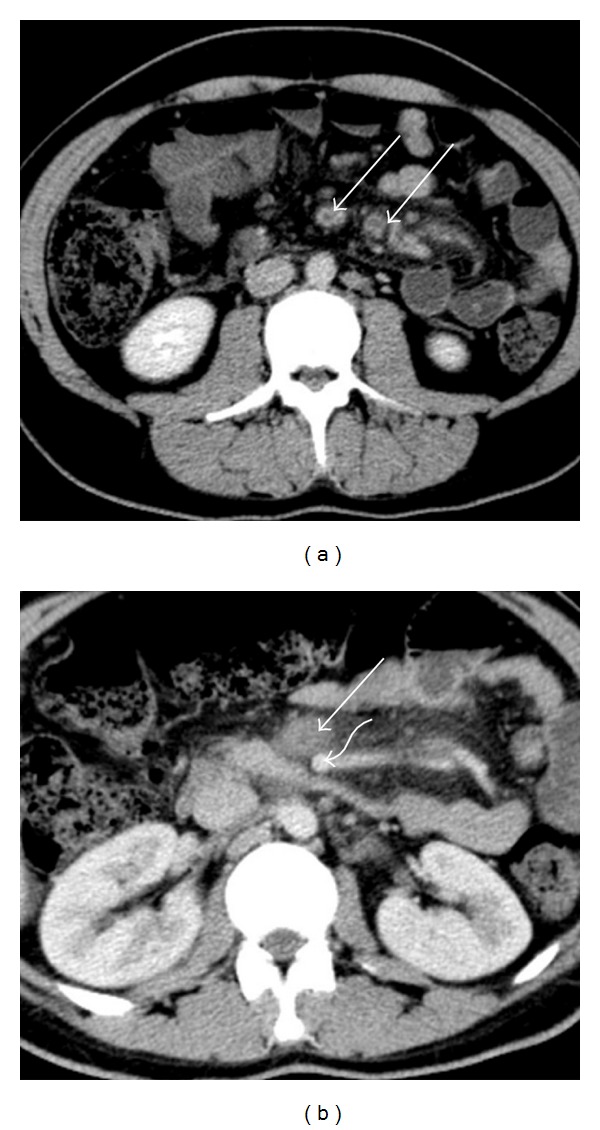
Axial contrast-enhanced CT images acquired at the level of the small-bowel mesentery root show nonenhancing low-attenuation thrombi within the lumen of the superior mesenteric vein tributaries (a) and superior mesenteric vein (b). In (b), the normal superior mesenteric artery is identified (curved arrow).

**Figure 3:**
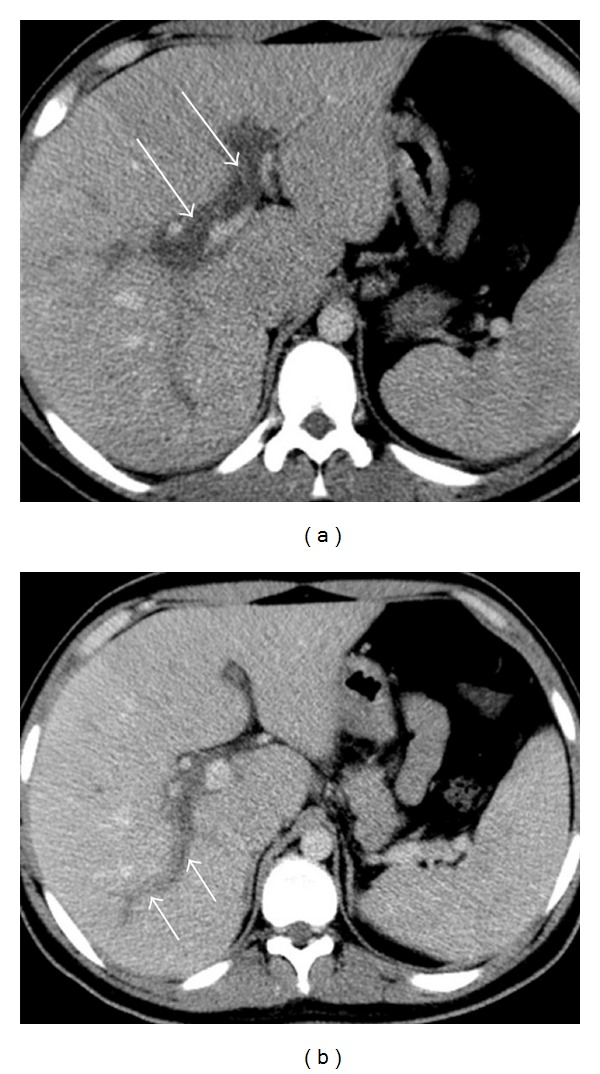
Axial contrast-enhanced CT images obtained through the midportion of the liver show thrombosis of the main portal branches (a) extending to the portal branch to the posterior segments of the right lobe.

Abdominal and pelvic contrast-enhanced CT study was performed. It revealed a dilated, hyperenhancing appendix, with surrounding mesenteric densification, indicative of acute appendicitis (Figure 1). There was an acute thrombus distending the lumen of the superior mesenteric vein and its tributaries, with inflammatory changes in the surrounding fat (Figure 2). There was also portal vein thrombosis (Figure 3). 

At laparotomy, acute appendicitis was confirmed and appendectomy was performed. After a two-week course of antibiotics and anticoagulation, the patient had clinical improvement with almost complete normalization of the laboratory tests results. He was discharged without symptoms.

**Figure 4:**
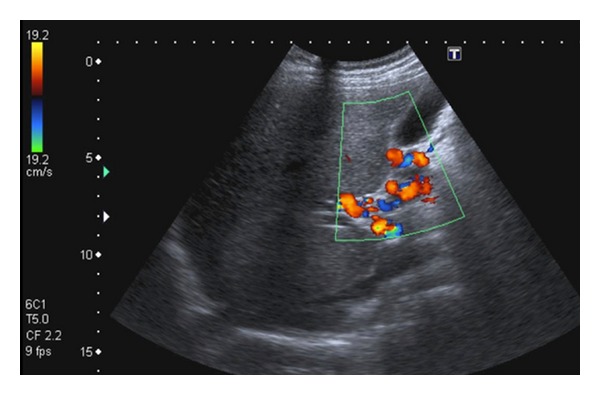
Color Doppler ultrasound image demonstrates multiple tortuous venous structures in the hepatic hilum, keeping with the cavernomatous transformation of the portal vein.

Two months later, the patient was in perfect clinical condition. Abdominal and pelvic evaluation with ultrasound demonstrated cavernomatous transformation of the portal vein (Figure 4). No other changes were seen. 

## 3. Discussion

Pylephlebitis refers to infective suppurative thrombosis of the portal vein and its branches. It is frequently associated with an intraabdominal inflammatory process. The most common intraabdominal causes of this entity are diverticulitis and appendicitis. Other described causes include necrotising pancreatitis, inflammatory bowel disease, haemorrhoidal disease, acute cholecystitis, and amoebic colitis [3, 4]. A recent abdominal surgery can also predispose to pylephlebitis [5].

The thrombus spreads from the small veins of the affected area to larger veins, leading to septic thrombophlebitis of the mesenteric vein and, eventually, of the portal vein [6]. This condition is associated with high morbidity and mortality because bowel ischemia and infarction may occur due to superior mesenteric vein thrombosis, and hepatic abscesses may complicate portal vein thrombophlebitis [7]. The clinical manifestations are often confusing and nonspecific. The patient may be asymptomatic, may have symptoms related to the primary intraabdominal process, or may present with an acute abdomen. Manifestations related to the thrombosis include abdominal pain due to bowel ischemia or jaundice and right upper quadrant pain due to liver involvement [8]. 

Modern imaging techniques like Doppler ultrasound and contrast-enhanced CT facilitate early diagnosis. Ultrasound may show portal vein thrombosis and signs of the primary abdominal inflammatory process, but its accuracy is limited by the interference of bowel gas. Contrast-enhanced CT scan can display intraabdominal processes like appendicitis and diverticulitis as well as mesenteric and portal vein thrombosis, liver abscesses, and bowel ischemia. 

Management of pylephlebitis consists of treating the primary septic process by using broad-spectrum antibiotics and adequate surgical intervention (appendectomy, colectomy, and abscess drainage) [1, 6]. The use of anticoagulation has been controversial. Full recovery is possible, although sometimes cavernomatous transformation of the portal vein and portal hypertension may emerge [9].

## 4. Conclusion

The combination of radiologic findings of a primary abdominal inflammatory process like appendicitis or diverticulitis and multiple thrombosis in the corresponding draining portal system veins is highly suggestive of pylephlebitis. A prompt diagnosis leads to early treatment and more successful clinical outcomes.
